# Use of Extracorporeal Membrane Oxygenation for Lung Cancer‐Related Massive Hemoptysis: A Case Report and Literature Review

**DOI:** 10.1002/rcr2.70662

**Published:** 2026-07-06

**Authors:** Yujie Zhou, Yifen Cai, Ziming Zhen, Lilong Qing, Shaojin Zhu, Lei Zha, Yusheng Cheng

**Affiliations:** ^1^ Department of Pulmonary and Critical Care Medicine The First Affiliated Hospital of Wannan Medical University Wuhu Anhui China; ^2^ Department of Thoracic Surgery The First Affiliated Hospital of Wannan Medical University Wuhu Anhui China; ^3^ Department of Pulmonary and Critical Care Medicine North District of the First Affiliated Hospital of Anhui Medical University Hefei China; ^4^ Anhui Public Health Clinical Center Hefei China

**Keywords:** ECMO, lobectomy, lung cancer, massive hemoptysis, pneumonectomy

## Abstract

Massive hemoptysis is a common, life‐threatening complication of lung cancer, and asphyxiation due to major airway obstruction is the leading cause of death. Extracorporeal membrane oxygenation (ECMO) can temporarily replace pulmonary gas exchange, stabilize acute respiratory failure, and provide a window for definitive therapy. This case report describes a patient with squamous cell carcinoma who developed catastrophic hemoptysis and successfully underwent right pneumonectomy under venovenous ECMO support, followed by uneventful recovery and discharge. In addition, a focused literature review was conducted to examine the role and optimal timing of ECMO as an adjunct to surgery for lung cancer‐related massive hemoptysis, offering practical insights for clinicians managing this critical condition.

## Graphical abstract

This case report describes a patient with squamous cell carcinoma who developed catastrophic hemoptysis and successfully underwent right pneumonectomy under venovenous ECMO support, followed by uneventful recovery and discharge. We show that VV‐ECMO can rapidly restore oxygenation and serve as a bridge to salvage surgery when BAE fails in asphyxiating hemoptysis. Heparin‐free VV‐ECMO is feasible during active airway bleeding, enabling safe thoracic surgery and that right pneumonectomy under VV‐ECMO is technically achievable in selected patients with resectable tumours. Early ECMO initiation and multidisciplinary collaboration are critical for successful management.

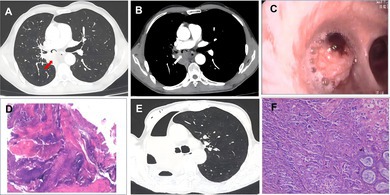

## 1 Introduction

Massive hemoptysis is a life‐threatening emergency and occurs in approximately 5% of lung cancer patients. Reported mortality rates vary from 6.5% to 38%. Deaths are primarily attributed to asphyxiation. Mortality from hemorrhagic shock is less common [1]. In lung cancer, catastrophic hemoptysis necessitates rapid stabilization and control of bleeding. Pharmacologic therapy and bronchial artery embolization (BAE) are widely regarded as first‐line interventions. However, thoracic surgery may be required after careful multidisciplinary evaluation. Although BAE is generally considered the preferred initial treatment for lung cancer‐related hemoptysis, failure is not uncommon in the setting of torrential bleeding or pulmonary arterial involvement. The failure rate was reported to be around 20% [2]. Salvayre et al. reported that more than one‐third of patients experienced rebleeding within 24 h after endovascular control. The 28‐day mortality was 25.3%. These findings underscore the poor prognosis associated with cancer‐related hemoptysis [3]. When embolization fails, surgery becomes an important salvage option if tumour stage and perioperative risk are acceptable [4].

Extracorporeal membrane oxygenation (ECMO), a key modality of extracorporeal life support, can partially or completely substitute for cardiopulmonary function. In patients with life‐threatening hemoptysis and central airway obstruction, ECMO offers rapid oxygenation, ventilatory support, and enhances procedural safety during bronchoscopic interventions, BAE, and definitive surgery treatment [5]. Here, we report a case of squamous cell carcinoma complicated by catastrophic hemoptysis and asphyxia, successfully rescued by right pneumonectomy under venovenous ECMO support. We also provide a focused review of the relevant literature.

## 2 Case Report

**FIGURE 1:**
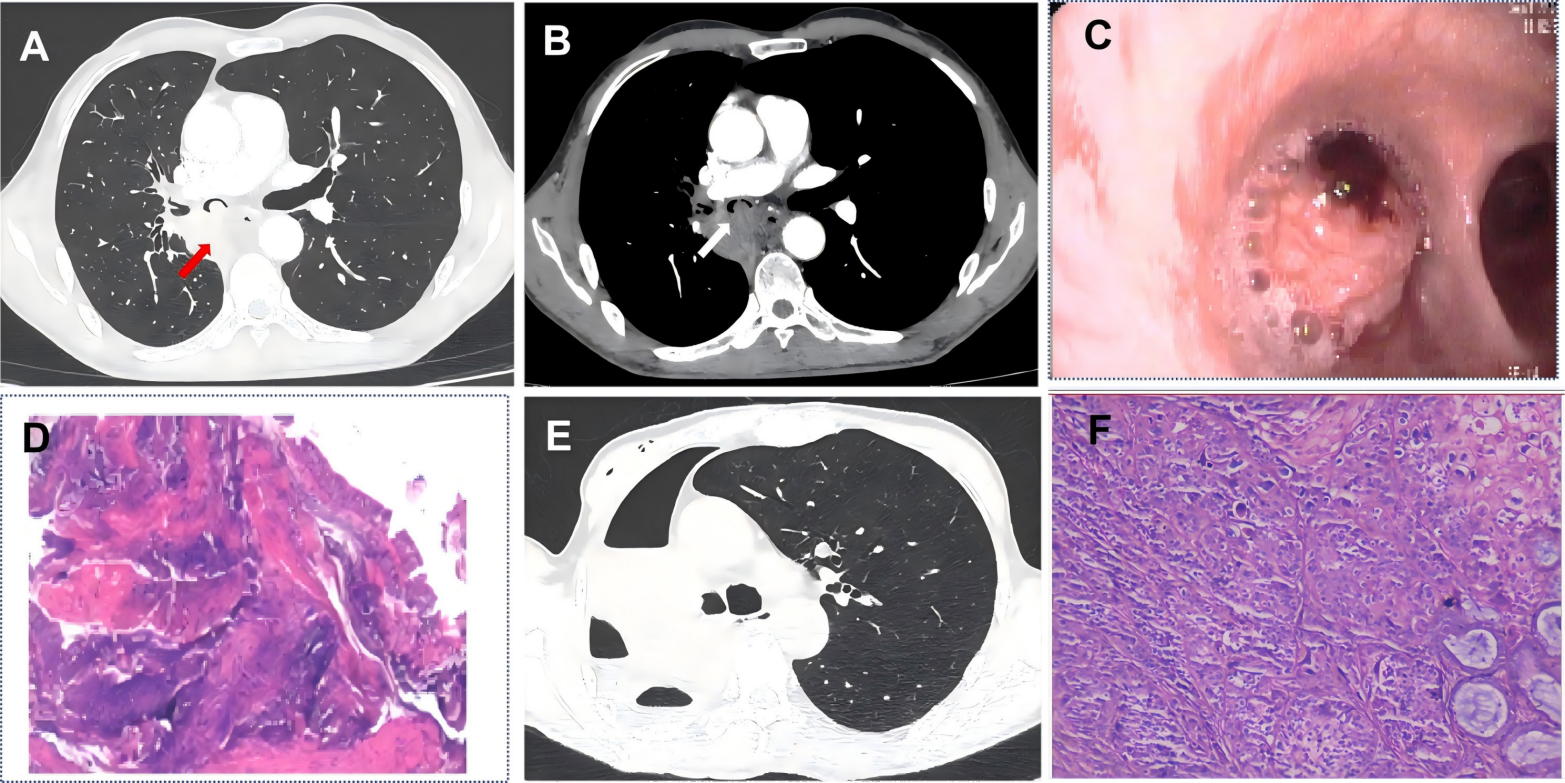
A patient with lung cancer presented with massive hemoptysis and underwent video‐assisted right pneumonectomy with mediastinal lymph node dissection on ECMO support. (A) Contrast‐enhanced CT images revealed right lower lobe mass (indicated by red arrow) and (B) endobronchial mass lesion (indicated by white arrow). (C) Bronchoscopy identified a friable, cauliflower‐like lesion occluding the right middle lobe bronchus. (D) Biopsy confirmed keratinizing squamous cell carcinoma. (E) CT images post video‐assisted right pneumonectomy with mediastinal lymph node dissection. (F) Post‐surgical pathology revealed invasive non‐keratinizing squamous cell carcinoma.

A 60‐year‐old man with a substantial smoking history presented with a 1‐month history of cough and sputum production, which progressed to blood‐streaked sputum during the week before admission. He had no history of hypertension or diabetes. On admission, he was hemodynamically stable and fully alert. Chest auscultation revealed coarse breath sounds over the right lung without rales. Contrast‐enhanced chest CT demonstrated a large right hilar mass obstructing the bronchus intermedius with resultant atelectasis of the middle and lower lobes and a small right pleural effusion (Figure 1A,B). Bronchoscopy showed a friable, cauliflower‐like lesion occluding the right middle lobe bronchus with brisk contact bleeding (Figure 1C). Histopathologic examination of bronchoscopic biopsies confirmed keratinizing squamous cell carcinoma (Figure 1D).

On hospital Day 6, the patient experienced sudden massive hemoptysis (> 200 mL in a single episode) accompanied by severe dyspnea, chest tightness, profound desaturation (nadir SpO_2_ 60%), and decreased level of consciousness, consistent with asphyxiating hemoptysis. Emergent endotracheal intubation was performed, and he was transferred to the respiratory intensive care unit. Bedside bronchoscopy for clots evacuation was unsuccessful. Despite mechanical ventilation with FiO_2_ 1.0, SpO_2_ remained approximately 60%. Immediate BAE was not feasible. Venovenous ECMO was therefore initiated with a pump speed of 3500 rpm, blood flow of 3.36 L/min, sweep gas flow of 2.5 L/min, and FiO_2_ 1.0. Mechanical Ventilation was adjusted to lung‐protective settings (FiO_2_ 0.60, PEEP 5 cm H_2_O, tidal volume 450 mL). Oxygenation improved rapidly, with SpO_2_ rising from 77% to 100%. Given the ongoing massive airway bleeding, heparin‐free VV‐ECMO was selected, with close surveillance for circuit thrombosis and a plan to introduce low‐dose heparin, if necessary, under strict monitoring of target activated clotting time (ACT) and activated partial thromboplastin time (aPTT).

Preoperative assessment classified the tumour as resectable stage IIIA lung cancer according to the TNM staging system. The patient's pre‐bleeding Eastern Cooperative Oncology Group performance status was 1, and he had no major comorbidities. He subsequently underwent video‐assisted right pneumonectomy with mediastinal lymph node dissection under venovenous ECMO support (Figure 1E). Pathology examination confirmed a centrally located invasive non‐keratinizing squamous cell carcinoma measuring 4.7 × 3.5 × 2.5 cm (Figure 1F). There was no perineural invasion or lymphovascular embolization. Bronchial and vascular margins were free of tumour. One of nine peribronchial lymph nodes harboured metastasis; all other sampled nodes were negative. ECMO was weaned and discontinued uneventfully on postoperative Day 3. No further hemoptysis occurred, and the patient recovered well and proceeded to planned adjuvant therapy.

## 3 Discussion

A PubMed and Embase search using combinations of ‘massive hemoptysis’, ‘life‐threatening hemoptysis’, ‘ECMO’, and ‘tumour’ or ‘cancer’ identified four comparable case reports in which patients with tumour‐related massive hemoptysis underwent successful salvage pulmonary resection supported by ECMO, and all patients survived to hospital discharge (Table 1). Common features included refractory hypoxemia or failure of transarterial embolization, prompting the use of ECMO to secure oxygenation and permit definitive surgery management.

**TABLE 1:** Four reported cases of cancer‐related massive hemoptysis underwent emergent salvage pulmonary resection under ECMO support.

Case	Gender	Age (years)	Diagnosis	Treatments	Outcome	References
1	Female	56	Invasive thymoma	Thymomectomy with removal of the involved tissues was urgently performed using the hemi‐clamshell approach and intrapericardial dissection	Cured	[6]
2	Female	28	Mediastinal hemangioma	Mediastinal hemangioma resection and right upper lobe sleeve resection	Cured	[7]
3	Male	61	Lung cancer in the right middle lobe	Video‐assisted thoracoscopic surgery was performed	Cured	[8]
4	Female	29	Bronchial hemangioma	Patient was performed the operation of right upper lung lobectomy and bronchial hemangioma	Cured	[9]

Asphyxiating hemoptysis can precipitate catastrophic respiratory and circulatory collapse within minutes, leaving a very narrow therapeutic window. When severe hypoxemia and airway obstruction persist, conventional strategies—pharmacologic haemostasis bronchoscopic measures, and even BAE—may be insufficient to prevent death. In this setting, ECMO offers distinct advantages by rapidly restoring oxygenation, maintaining gas exchange, and improving procedural safety during critical airway and hemostatic interventions [5]. Anticoagulation management in the presence of active airway bleeding is particularly challenging; heparin‐sparing approaches, including temporary discontinuation or low‐dose regimens, are increasingly used to balance thrombosis and bleeding risks in closely monitored patients [10].

Beyond cancer‐related hemoptysis, ECMO has been employed as rescue or bridging support in a broad range of scenarios involving massive hemoptysis and high‐risk interventional pulmonology procedures. Reported indications include complex tumour debulking, airway stent placement, and pulmonary resections performed under ECMO support [5, 11]; embolization of vascular malformations; VV‐ECMO‐assisted management of tuberculosis‐related respiratory failure during transcatheter arterial embolization [12]; bilateral bronchial haemorrhage managed with VV‐ECMO and staged bronchoscopic clearance [13]; prolonged heparin‐free VV‐ECMO enabling thoracic surgery and endovascular interventions during active haemorrhage [10]; risk mitigation during rigid bronchoscopy for tracheal stenting [14]. Collectively, these experiences suggest that ECMO can create a crucial window to achieve definitive haemostasis and secure airway control in the context of life‐threatening hemoptysis. Successful implementation depends on advanced institutional resources and close multidisciplinary collaboration among intensivists, oncologists, thoracic surgeons, and perfusion specialists.

In conclusion, in lung cancer–related massive hemoptysis, rescue VV‐ECMO can stabilize gas exchange, facilitate definitive pulmonary resection, and offer a potential pathway to meaningful recovery.

## Funding

This research received support from the Key Research Project of Anhui Provincial Health Commission (AHWJ2023A10132).

## Consent

The authors declare that written informed consent was obtained for the publication of this manuscript and accompanying images using the form provided by the Journal.

## Conflicts of Interest

The authors declare no conflicts of interest.

## Data Availability

The data that support the findings of this study are available on request from the corresponding author. The data are not publicly available due to privacy or ethical restrictions.
